# The effect of pulsed current on the shear deformation behavior of Ti-6Al-4V alloy

**DOI:** 10.1038/s41598-018-32857-6

**Published:** 2018-10-03

**Authors:** Zhiyong Zhao, Guofeng Wang, Hongliang Hou, Yanling Zhang, Yaoqi Wang

**Affiliations:** 1 National Key Laboratory for Precision Hot Processing of Metals, Harbin Institute of Technology, Harbin, 150001 China; 2 Beijing Aeronautical Manufacturing Technology Research Institute, Beijing, 100024 China

## Abstract

Pulse current-assisted forming is a new technology to improve the plastic deformability of titanium alloy. In this work, Shearing tests of Ti-6Al-4V alloy were conducted using hat-shaped specimens under pulsed current (electroplastic shearing) and constant temperature (isothermal shearing). The actual deformation in shear zone of electroplastic shearing was larger than that of isothermal shearing. The shear load is also decreased by the pulsed current. Microstructure variation in the shear zone was investigated by scanning electron microscopy. An evident straight shear band was observed in the electroplastic shearing specimens. The deformation model of shear zone was established. Intracrystalline deformation was markedly easier for the grains with the pulsed current and induced larger deformation of the grains along the shear direction. Microcracks were observed in the shear zone of isothermal shearing, but none were found in the shear zone of electroplastic shearing. Evident crack healing was found in the crack tip of the shear zone of electroplastic shearing.

## Introduction

Shear testing is a well-known approach to study the mechanical behavior of metals. In practical applications, materials are often dynamically loaded directly in shear (punching, machining, and impact)^1–4^. At present, most studies have focused on dynamic shear behavior and the formation mechanism of adiabatic shear bands^5–7^, where as the shear performance of material itself, such as shear strength and shear plastic deformability, have been rarely investigated.

Titanium and titanium alloys are the design choice for aerospace, biomedical, and other applications because of an advantageous combination of low density, good mechanical properties, high corrosion resistance, and biocompatibility^8–11^. Ti-6Al-4V is the most commonly used titanium alloy, and it is often selected in studying the formation of adiabatic shear bands^12–14^. Considerable research has been conducted on adiabatic shear banding and failure in this material. J. Peirs *et al*. and Liu *et al*. studied the effect of microstructure and strain rate on shear behavior and the correlation of the shear band with fracture of Ti-6Al-4V alloy using hat-shaped specimens and split Hopkinson bar^4,15^. YaBei Gu *et al*. investigated the dynamic shear behavior of hot isostatically pressed Ti-6Al-4V^7^. Chao Zheng *et al*. analyzed the dynamic compression properties and sensitivity of the formation of the adiabatic shear band of Ti-6Al-4V alloy with equiaxed and bimodal microstructures, respectively^16^.

Machining and forming are difficult for the Ti-6Al-4V alloy at room temperature because of several properties. The poor shear plastic deformability of the Ti-6Al-4V alloy is one of the major causes inducing failure during forming. Pulsed current has been studied by numerous researchers and is considered an effective method to improve the performance of metal plastic forming^17,18^. This approach is highly effective in improving plastic deformation and decreasing deformation resistance in rolling, bending, and drawing technologies^19–22^. Additionally, the ultra-fast annealing and avoiding crack propagation induced by electropulsing are also detected in hybrid mixed double-sided incremental forming and electrically assisted wire drawing process^23,24^. However, the influence of pulsed current on pure shear deformation has rarely been studied.

This study focused on investigating the pulsed current while performing shear deformation with hat-shaped specimens made of Ti-6Al-4V alloy. To this end, the material microstructure and the deformation mechanism during shearing deformation are studied while assisting the Ti-6Al-4V with an electropulsing field and also with a conventional thermal treatment. Finally, the tip area of each specimen are explored to state the influence of both thermal sources in the crack evolution at shear zone, which, ultimately will describe the type of fracture failure.

## Materials and Experimental Method

**Figure 1:**
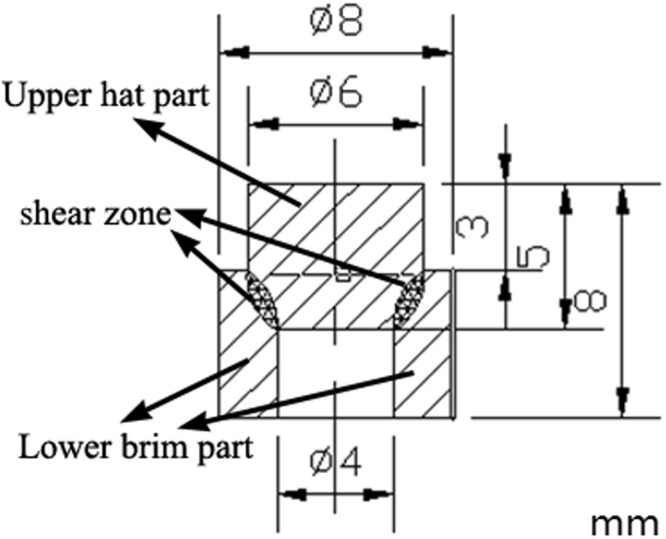
Hat-shaped specimen using in shearing test.

**Figure 2:**
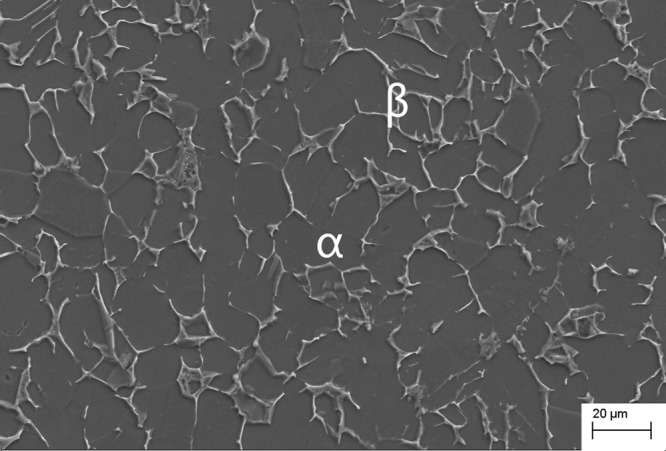
The original microstructure of Ti-6Al-4V alloy.

The axi-symmetric hat-shaped specimen was selected to employ the experiments, where shear strains are concentrated in a narrow zone, as shown in Fig. 1. The specimen can be divided into three regions: the upper hat part, the lower brim part and the shear zone where high shear strain develop. The specimens are carefully machined out of a standard extruded Ti-6Al-4V bar (chemical component: 6.66% Al, 5.13% V, 0.21% Fe, 0.03% Mo, balance Ti). The alloy consists of a majority of equiaxed hexagonal (HCP) α-phase with grain size of 20~30 μm and a finely dispersed cubic (BCC) β-phase. The original microstructure of experimental material was shown in Fig. 2 which observed by SEM.

**Figure 3:**
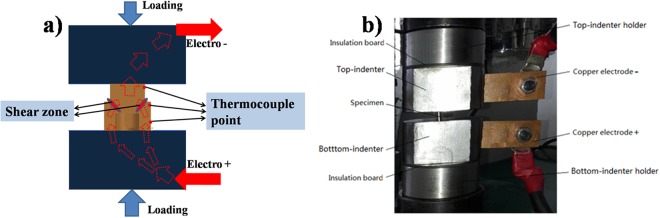
Detailed view of electroplastic shearing tests. (**a**) Schematic diagram; (**b**) experiment diagram.

The shearing tests are conducted under the conditions of applying pulsed current (electroplastic shearing) and constant temperature (isothermal shearing). Figure 3 shows the detailed view of electroplastic shearing tests. The hat-shape specimen was placed between the two indenters which were connected with electrodes. The path of current conduction was indicated with arrows in Fig. 3(a). The electricity enters the top copper electrode, passes through the indenters and specimen and exists at the lower copper electrode. The electrical parameters of pulsed current including discharge voltage, frequency, root-mean-square current (RMS), amplitude of the currency and duration of single current pulse were all monitored by an electrical Hall Effect sensor connected to an oscilloscope. The discharge voltage applied in experiments is 40 V, 50 V and 60 V, respectively, with the frequency of 300 HZ.

Temperature of the position located at the upper hat part, the lower brim part and the shear zone, as shown in Fig. 3, was concurrently measured by a storable K type surface contact thermocouple and the recorded temperature can be analyzed in the real-time temperature–time evolution monitoring system. Only 500 N pre-load was applied in order to ensure well electrical conductivity between hat-shape specimen and indenters in the temperature test.

**Figure 4:**
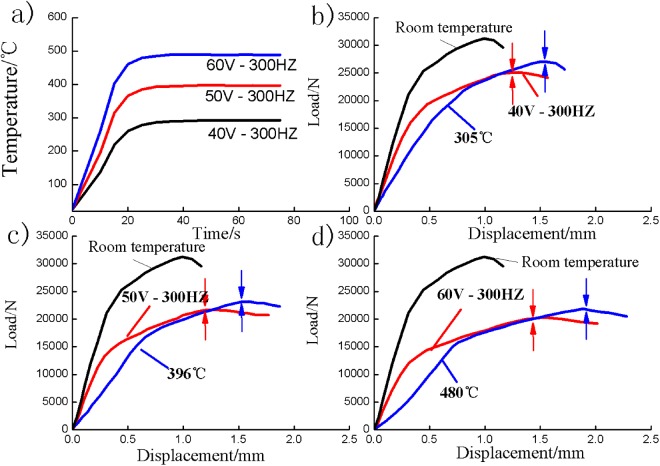
Result of electroplastic shearing and isothermal shearing. (**a**) Temperature – time curves of shear zone; load–displacement curves of: (**b**) 40 V and 305 °C; (**c**) 50 V and 396 °C; (**d**) 60 V and 480 °C.

According to the temperature results, as displayed in Table 1 and Fig. 4(a), The temperature of the shear zone increased rapidly after application of the pulsed current to the hat-shaped specimen and reached stable levels of 305 °C, 396 °C, and 480 °C within 30 s. The shearing test started at 45 s after the pulsed current was applied, so the temperature was distributed evenly throughout the shear zone. The speed of shearing tests is 80 mm/min. Shearing test was determined to be finished as the peak shear load reduced by 10%.

**Table 1:** Current density and temperature results in electroplastic shearing.

Voltage/V	*j*_*m*_/A · mm^−2^	*j*_*e*_/A · mm^−2^	*T*_up_/°C	*T*_shear_/°C	*T*_low_/°C
40	45	4.7	212	305	223
50	57	6.2	306	396	315
60	68	6.9	395	480	404

*j_m_: Amplitude of current density.

*j_e_: Root-mean-square (RMS) current density.

*T_up_: Measured temperature of the upper hat part.

*T_shear_: Measured temperature of the shear zone.

*T_low_: Measured temperature of the lower brim part.

To distinguish the thermal and athermal effects of pulsed current in shear deformation of Ti-6Al-4V alloy, another group of shearing tests were performed at isothermal condition, the furnace temperature is in agreement with the measured temperature of shear zone at the condition of applying pulse current. In addition, the shearing test at room temperature without pulse current was also conducted. Moreover, all group specimens, including both electroplastic shearing and isothermal shearing, are set as three parallel tests for obtaining average results in order to conduct the strict/precision standard in the experiment avoiding the accidental error.

After shearing tests, the hat-shape specimens were axially sectioned and prepared for metallographic examination. The chemical attack for Ti-6Al-4V alloy was 30 mL HF + 10 mL HNO_3_ + 30 mL H_2_O and a ZEISS SIGMA 500 scanning electron microscope (SEM) was used to observe the microstructures. Electron back scattered diffraction (EBSD) technique was used to examine the microstructure of the specimens, which was performed on a ZEISS-EVO18 SEM, and the scanning step size was 0.3 μm and the results were analyzed using HKL Channel 5 software. The distribution of alloying elements in the micro-crack was examined by electro-probe microanalyzer (EPMA).

## Result and Discussion

### Shear plastic deformability

The results of amplitude of current density *j*_*m*_, root-mean-square current density *j*_*e*_, which were measured by an oscilloscope are displayed in Table 1. The values of *j*_*m*_ and *j*_*e*_ are both increased with increasing of applied voltage. The shear load and displacement were recorded during experiments, and the load–displacement curves are shown in Figs 4(b–d) and S1. By contrast with shearing test at room temperature, the shear load decreased and the shear deformation increased with increasing voltage and temperature, indicating that high voltage and temperature helped in improving the shear plastic deformability of the Ti-6Al-4V alloy. However, the shear load and total deformation of isothermal shearing were larger than those of electroplastic shearing. The shear load increased rapidly with increasing shear deformation at the early stages of electroplastic shearing and then rose to the maximum value steadily. The shear deformation of isothermal shearing exceeded that of electroplastic shearing at the stage of rapid increase in shear load. The point of the maximum load is marked by two opposite arrows in Fig. 4(b–d). Compared with isothermal shearing, electroplastic shearing consumed larger shear deformation from the maximum load to the end.

**Figure 5:**
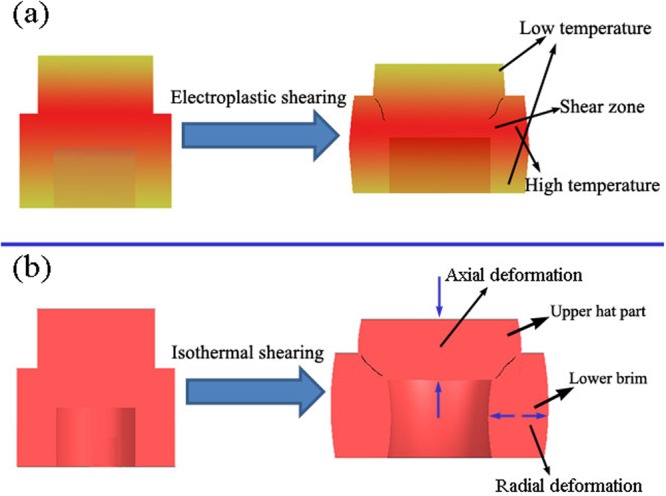
Deformation diagrams of hat-shape specimen in: (**a**) electroplastic shearing and (**b**) isothermal shearing.

**Figure 6:**
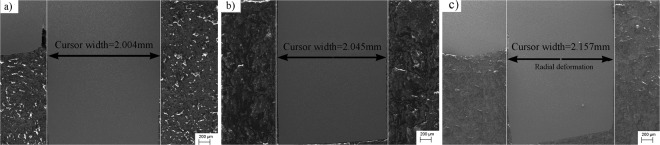
Deformation of lower brim in. (**a**) Room temperature shearing; (**b**) 60 V electroplastic shearing; (**c**) 480 °C isothermal shearing.

Numerous researchers concluded that the pulsed current can improve the plasticity of metal materials^25,26^. However, the deformation of electroplastic shearing is smaller than that of isothermal shearing. We believe that this difference is due to non-uniformity of the temperature distribution in the sample in electroplastic shearing. According to Joule’s law, the temperature of the hat-shaped specimen will inevitably increase due to long-term application of electropulses. However, the heat generated by the upper hat part and lower brim will conduct to indenters. Therefore, the temperature of the shear zone was higher than that of the upper hat part and lower brim, as confirmed by the measured temperature (Table 1). The temperature of every part of the specimen in isothermal shearing was distributed uniformly, leading to higher temperature of the upper hat part and lower brim in isothermal shearing than in electroplastic shearing. Figure 5 shows the deformation diagrams of the hat-shaped specimens in electroplastic shearing and isothermal shearing. The entire specimen in isothermal shearing, including the upper hat part and lower brim, received larger plastic deformation, but the deformation was mainly concentrated at the shear zone in electroplastic shearing. Figure 6 shows that the deformation of the lower brim in 480 °C isothermal shearing was more evident than that in 60 V electroplastic shearing and room temperature shearing. The thickness of the brim part of the original sample was 2 mm. After shearing tests, the thickness of the brim part of 60 V electroplastic shearing and room temperature shearing specimens changed little, this suggested that little deformation was occurred at the brim part. However, the thickness increased to 2.157 mm after isothermal shearing. Evidently, larger radial deformation of the lower brim was observed in isothermal shearing. Therefore, the total deformation of the hat-shaped specimens in isothermal shearing was larger than that in electroplastic shearing. On the other hand, the uneven deformation of each part of the hat-shaped specimens also leads to the inconsistency of the slope of load–displacement curves at initial stage. The total deformation of the hat-shaped specimens is larger with temperature increasing and better distribution under the same load.

The peak load refers to the maximum value of load before crack generation in the shear zone, which can reflect the influence of the pulsed current on the shear plastic deformability of the Ti-6Al-4V alloy. All peak loads in electroplastic shearing were smaller than those in isothermal shearing; thereby illustrating that shear deformation resistance could be decreased by the electroplasticity of the pulsed current. Annealing caused by pulsed current is one possible thermal effect that can effectively promote the dynamic recovery, which is connected with the dislocation annihilating^25,26^. Aiding pulsed current, dislocation pile-up in deformed materials is eliminated, and the dislocations are parallel to the direction of electron movement. The decrease in dislocation density is the main explanation for the low shear deformation resistance in electroplastic shearing^27^ (Fig. S2).

### Microstructure variation in the shear zone

**Figure 7:**
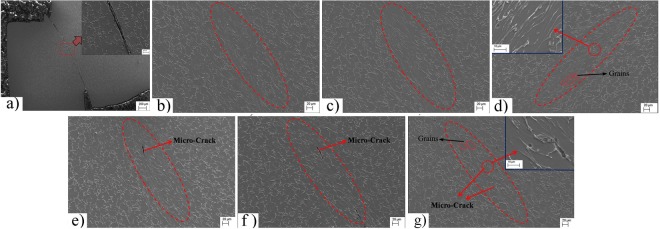
Microstructures of shear zone. (**a**) Room temperature; (**b**) 40 V; (**c**) 50 V; (**d**) 60 V; (**e**) 305 °C; (**f**) 396 °C; (**g**) 480 °C.

The morphology of Shear deformation zone tested at room temperature was shown in Fig. 7(a). The crack runs through the whole shear deformation zone, and the grain shape in the deformation zone is almost unchanged, indicating that little plastic deformation occurred in the room temperature shearing test. This is due to the poor plastic deformation ability of Ti-6Al-4V alloy at low temperature and high strain rate, the cracks is easily produce in the grain boundary and propagate rapidly under the externally applied stress (Fig. S3). The microstructures of the shear zone tested with pulsed current are shown in Fig. 7(b~d). Evident straight shear bands distinguished from the matrix by boundaries were observed in the electroplastic shearing specimens. The grains in the shear zone were elongated along the shearing direction under shear stress and hydrostatic pressure. The aspect ratio of grains increased with increasing voltage. No cracks were observed in the shear deformation zone. By contrast, in isothermal shearing (Fig. 7e~g), smaller deformation occurred in grains of the shear zone, and the edge of the shear band and matrix was difficult to distinguish. Although a portion of equiaxial grains changed to long strips, the aspect ratio was markedly smaller than that in electroplastic shearing. The aspect ratio of grains as marked in Fig. 7(c) is only 0.2~0.3, but in Fig. 7(f), the aspect ratio of marked grains is 0.4~0.5. In addition, grains close to fracture cracks exhibited larger deformation than those in the middle of the shear zone. Several small cracks were also found in the shear deformation zone, as shown in Fig. 7(d~f).

**Figure 8:**
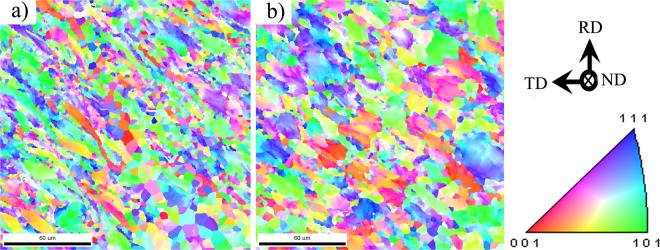
EBSD orientation maps of shear zone. (**a**) 60 V electroplastic shearing; (**b**) 480 °C isothermal shearing.

In order to use EBSD to further analyze the microstructure evolution of shear zone, the specimens tested with 60 V electroplastic shearing and 480 °C isothermal shearing was first heat-treated at the temperature of 700 °C for 30 min, which is the conventional annealing treatment (Fig. S4). The purpose of heat-treatment is eliminating residual stress of specimens and acquired enough orientation data in EBSD measurement. The results of EBSD were shown in Fig. 8. A lot of recrystallized fine grains with size of 5~10 μm are achieved in the shear zone of 60 V electroplastic shearing specimen. The large plastic deformation increases the occurrence of static recrystallization, even though the annealing temperature is relatively low. However, the grain size and shape in shear zone of 480 °C isothermal shearing change little during the annealing process, only a few fine grains precipitate out in grain boundary. In addition, the difference of grains aspect ratio can be seen more clearly from Fig. 8(a,b). From the results, it could be confirmed that the actual deformation of the shear zone in electroplastic shearing was larger than that in isothermal shearing. The shear plastic deformability of the Ti-6Al-4V alloy could be improved by the pulsed current, and the improvement of plastic deformation and reduction of deformation resistance not only because of Joule heating but also because of the pulsed current being applied during plastic deformation. It can be said that current-induced annealing and thermal recovery takes place when a pulsed current is applied to the specimen during shear plastic deformation. This could be related to the electric current enhancing the atomic diffusion, reducing dislocation pile-up and tangling and avoiding the local stress concentration^25–27^.

## Deformation Mechanism of Electroplastic Shearing

**Figure 9:**
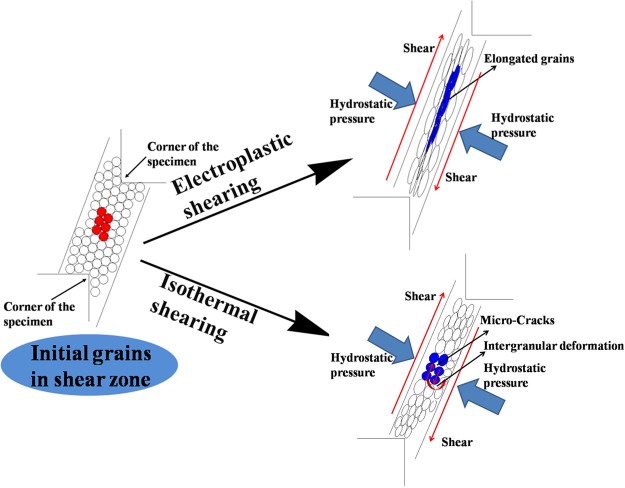
Sketch of grains deformation in the shear zone.

Figure 9 illustrates the deformation of grains in the shear zone. The grains initially appeared as circles. Distortion occurred in the grains because they were grinding against each other under shear stress and hydrostatic pressure. Intracrystalline and intergranular deformations both occurred during the shearing test to accommodate the large imposed strain. Intracrystalline deformation induced a change in grain shape from equiaxial to long strips, but the sliding and rotation between grains caused by intergranular deformation made the long strip grains parallel to the shearing direction. Intergranular deformation was more difficult because the strength of the grain boundary was higher than that in the interior of the grain^28^, and the grains in the shear zone were in contact with one another and presented an interlocking shape.

**Figure 10:**
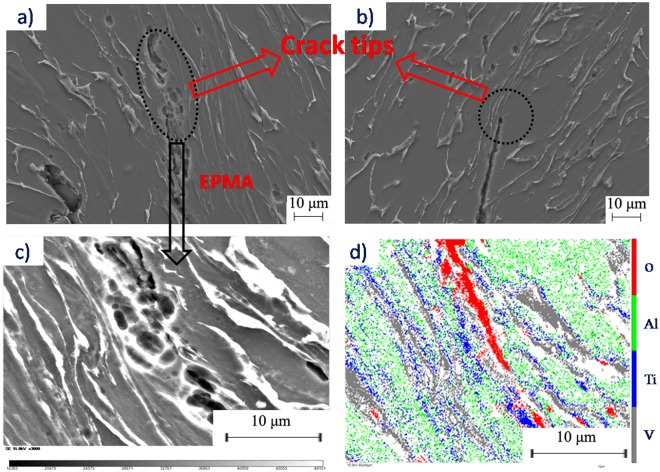
Morphology and analysis of crack tip. (**a**) Crack tip in electroplastic shearing; (**b**) crack tip in isothermal shearing; (**c**) crack welding in electroplastic shearing; (**d**) distribution of alloying elements tested by EPMA.

Dislocation pile-up and tangling may occur inside grains because of the large strain and strain rate. The pulsed current promoted dislocation movement and induced an orderly arrangement of dislocations. The dislocation density and internal stress of grains decreased because of the pulsed current. Therefore, intracrystalline deformation more easily occurred in electroplastic shearing compared with isothermal shearing, and the grains in electroplastic shearing could achieve large deformation. This outcome led to a larger aspect ratio of grains in electroplastic shearing than in isothermal shearing. More intergranular deformation was needed in isothermal shearing, whereas intracrystalline deformation could not accommodate the imposed shear strain. However, the intergranular deformation is difficult due to the high strain rate, the large grain size and the relatively low deformation temperature. Microcracks along the grain boundary formed when intergranular deformation exceeded its limitation. Microcracks may also be generated in electroplastic shearing, but none were observed in the shear deformation zone of electroplastic shearing. On one hand, the results of Fig. 7 show that grains in shear zone of electroplastic shearing can obtain larger plastic deformation than that of isothermal shearing; on the other hand, micro-cracks can be healed and inhibited instantaneously with the help of pulsed current. Resistance in the microcracks increased instantaneously, and the temperature also increased rapidly with the pulsed current. Microcracks were pressed together by the hydrostatic pressure perpendicular to the shear direction and then welded together by heat generated by the pulsed current. Various studies investigated crack healing techniques using pulsed currents^29–31^, and their results showed that the generation of Joule heating and hydrostatic pressure between crack on both sides are two important factors affecting the process of crack healing^32,33^. As shown in Fig. 10, the tip of the fracture crack in electroplastic shearing was blunter than that in isothermal shearing. Evident crack welding was found with further observation (Fig. 10(c)). The results of EPMA (Fig. 10d)) showed that oxidation occurred at high temperatures during crack welding. The crack healing of the pulsed current was also the main cause of large shear deformation consumed from the maximum load to the end of the electroplastic shearing test.

## Conclusion

The effect of the pulsed current on the shear plastic deformability of the Ti-6Al-4V alloy was studied via electroplastic shearing and isothermal shearing tests using hat-shaped specimens. The shear plastic deformability of Ti-6Al-4V alloy was improved by the pulsed current because of its thermal effect and electroplasticity. The grains’ plastic deformation is more significant due to current-induced annealing and thermal recovery, which was caused by the pulsed current. Intracrystalline deformation was markedly easy for the grains in electroplastic shearing, and large deformation occurred in those grains. Therefore, an evident shear band was found in the shear zone of electroplastic shearing. Microcracks that were generated during shearing could be healed by Joule heating and high hydrostatic pressure. Microcrack healing was another main cause of the improvement in shear plastic deformability in electroplastic shearing.

## Electronic supplementary material

Supplementary materials

## Data Availability

Te datasets are available upon reasonable request.
