# Supplementary material for: The effect of pulsed current on the shear deformation behavior of Ti-6Al-4V alloy
Source: Sci Rep. 2018 Oct 3;8:14748. doi: 10.1038/s41598-018-32857-6 (PMC6170433; doi:10.1038/s41598-018-32857-6)
Supplement: Supplementary file 1 — Supplementary materials [file 41598_2018_32857_MOESM1_ESM.docx]

**The effect of pulsed current on the shear deformation behavior of Ti–6Al–4V alloy**

Zhiyong Zhao^1,2^, Guofeng Wang^1*^, Hongliang Hou^2^, Yanling Zhang^2,^ Yaoqi Wang^2^

1 National Key Laboratory for Precision Hot Processing of Metals, Harbin Institute of Technology, Harbin 150001, China

2 Beijing Aeronautical Manufacturing Technology Research Institute,

Beijing 100024, China

*Correspondingauthor: Guofeng Wang，E-mail address: [gfwang@hit.edu.cn](mailto:gfwang@hit.edu.cn) and Hongliang Hou, E-mail address: hou_hl@163.com

[**Supplementary**](http://www.nature.com/srep/publish/guidelines#supplementary-info) **materials**





**Fig. S1** deviations of: (a )maximum load and (b) deformation displacement

All group specimens, including both electroplastic shearing and isothermal shearing, are set as three parallel tests for obtaining average results in order to conduct the strict/precision standard in the experiment avoiding the accidental error. **Fig. 4 (b-d)** show the average results of maximum load and deformation displacement, and the deviations of maximum load and deformation displacement were shown in **Fig. S1**. Although errors were existed for the experimental results under the same condition, the regularity of the results are consistent.

**
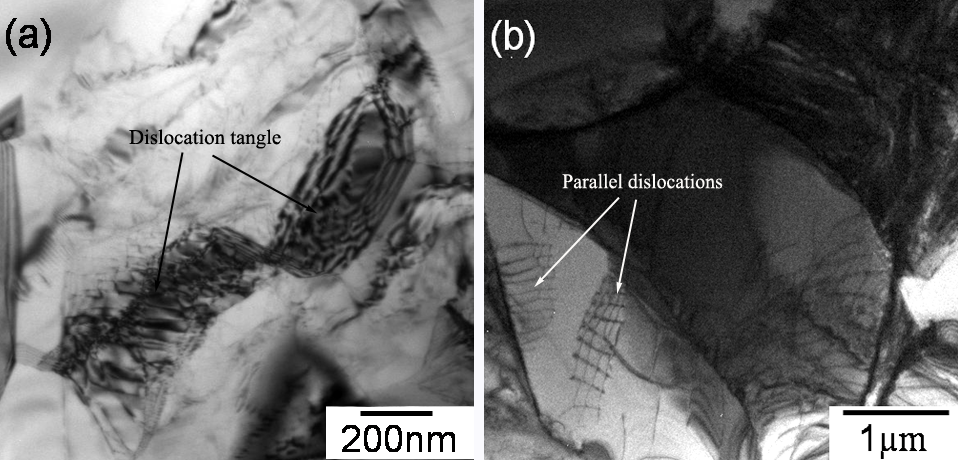
**

**Fig. S2** TEM micrograph showing the dislocation morphology of (a) compressed without electropulsing and (b) compressed with electropulsing [27]

In our previous works [27], with the aid of electron wind force, the elimination of dislocation pile-up in deformation materials and the dislocations climbing parallel to the movement direction of the electrons were observed in the compression tests of Ti-6Al-4V alloy. The dislocation density was also decreased with the help of pulses current.


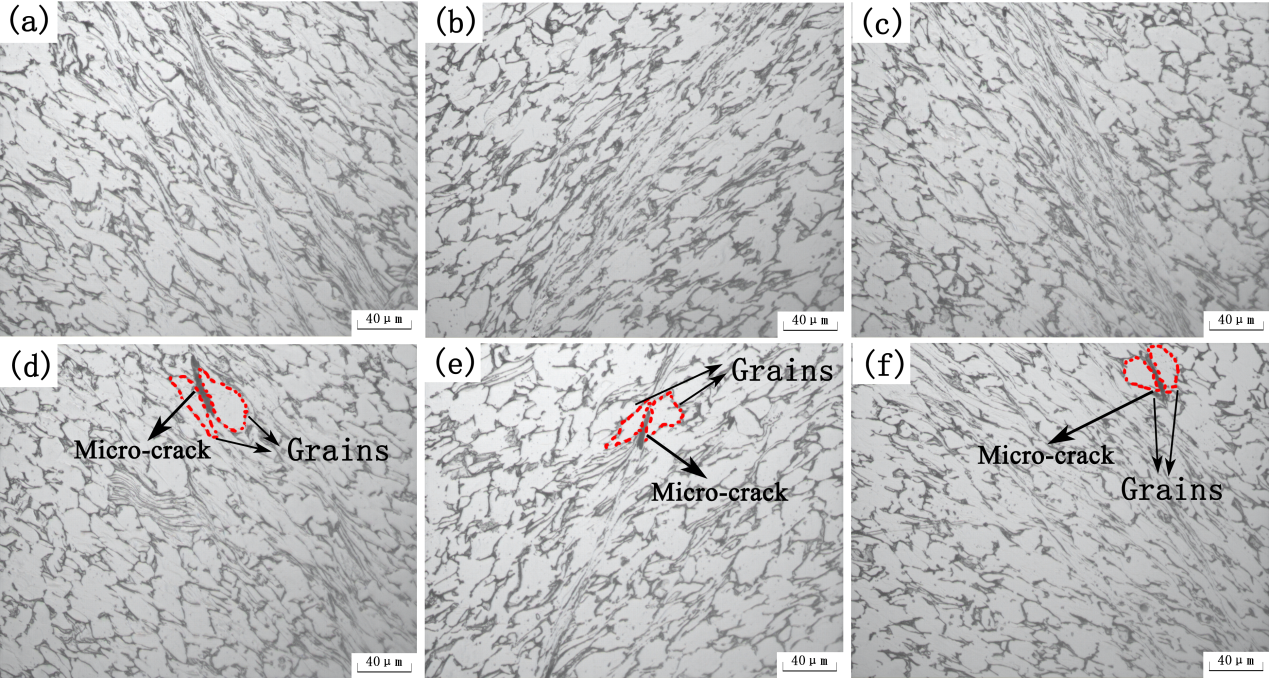
**Fig. S3** Optical metallography pictures of shear zone: a) 40V; b) 50V; c) 60V; d) 305 ℃; e) 396 ℃; f) 480 ℃;

**Fig. S3** shows the optical metallography pictures of shear zone, there was no cracks observed in samples of electroplastic shearing, but clearly cracks were observed in samples of isothermal shearing, and two separate grains were observed on either side of the cracks. From the optical metallography pictures, we can deduce that cracks grow and expand along the grain boundary. Moreover, from **Fig. S3**, the larger deformation in shear zone of electroplastic shearing that that of isothermal shearing was more clearly, and the deformation of grains in shear zone of electroplastic shearing was also larger than that of isothermal shearing.





**Fig. S4** temperature curve of annealing treatment

The samples were put into a furnace and heated from room temperature to 700 ℃ within 30 min, and keep 700 ℃ for 30 min, then take out the sample from the furnace and cooling in the air.
